# Pitman Clemens Spencer, M.D.

**Published:** 1860-05

**Authors:** 


PITMAN CLEMENS SPENCER, M.D.—In the death of Dr. Spencer, which occurred last January, at his residence at Petersburg, Virginia has lost one of its brightest and most exemplary surgical ornaments, a man of great purity of character, an excellent citizen, and an eminently popular practitioner; one whose name and fame are intimately associated with the medical history of his native State. He was born in Charlotte County, in July, 1793, and was, consequently, at the time of his decease, in the sixty-seventh year of his age. His early educational advantages were, it would seem, very slender; but by energy and industry in after-life he surmounted his deficiencies, and eventually became highly intelligent, not only in his profession, but in general literature and science. Having studied medicine in the office of his brother, he subsequently attended lectures in the University of Pennsylvania, in the time of Wistar and Physick, obtaining the honors of the doctorate in 1818. In 1827 he visited Europe, and, on his return in 1830, finally settled at Petersburg, where he remained up to the time of his death, in the enjoyment of a large practice, his surgical reputation attracting patients from all sections of Virginia, and the eastern and northern parts of North Carolina. He was often called to cases at a great distance. His great forte was in surgery, in which his success was uncommonly flattering. His skill as an operator was striking. He handled the knife with consummate ability, and it was seldom that it deceived him. As a lithotomist, his exploits were remarkable. In 1855, he communicated to the writer of this notice the particulars of twenty-four cases upon which he had operated up to that period, with a loss of only two, or in the proportion of one to twelve. Subsequently he cut five other cases without a single loss. Of such a result any operator might justly be proud. He preferred the bilateral method, with Dupuytren's lithotome caché, having, doubtless, contracted a fondness for the procedure during his sojourn in Paris, where he devoted much attention to vesical diseases and operations. Of lithotripsy he entertained no very favorable opinion, and never practiced it upon the living subject.

But, although Dr. Spencer knew how to use the knife, he never resorted to it when it was possible to avoid it. He had great confidence in the resources of nature, and always felt a just pride in his conservatism, well knowing that it is much more of an honor to preserve a limb than to lop it off. In him were happily blended the good qualities of the physician and the manual dexterity and unflinching eye of the surgeon.

The career of such a man as Dr. Spencer furnishes a bright example for the imitation of the young men of the profession. It shows what, despite of early trials and difficulties, may be achieved by honest industry, and a resolute aim in life, determined to surmount all obstacles, and to make everything bend to the accomplishment of its purposes. He was a gentleman of enlarged and liberal mind: and to him may justly be applied the words of Terence: "Homo sum; humani nihil a me alienum puto."

